# Identification of low-overpressure interval and its implication to hydrocarbon migration: Case study in the Yanan sag of the Qiongdongnan Basin, South China Sea

**DOI:** 10.1371/journal.pone.0183676

**Published:** 2017-09-21

**Authors:** Qinghai Xu, Wanzhong Shi, Yuhong Xie, Zhenfeng Wang, Xusheng Li, Chuanxin Tong

**Affiliations:** 1 Key Laboratory of Tectonics and Petroleum Resources (China University of Geosciences), Ministry of Education, Wuhan, China; 2 Faculty of Earth Resources, China University of Geosciences, Wuhan, China; 3 Zhanjiang Company, CNOOC, China; IIT(ISM) Dhanbad, INDIA

## Abstract

The Qiongdongnan Basin is a strongly overpressured basin with the maximum pressure coefficient (the ratio of the actual pore pressure versus hydrostatic pressure at the same depth) over 2.27. However, there exists a widespread low-overpressure interval between the strong overpressure intervals in the Yanan Sag of western basin. The mechanisms of the low-overpressure interval are not well understood. Three main approaches, pore pressure test data and well-log analysis, pressure prediction based on the relationship between the deviation of the velocity and the pressure coefficients, and numerical modeling, were employed to illustrate the distribution and evolution of the low-overpressure interval. And we analyzed and explained the phenomenon of the low-overpressure interval that is both underlain and overlain by high overpressure internal. The low-overpressure interval between the strong overpressure intervals can be identified and modelled by drilling data of P-wave sonic and the mud weight, and the numerical modeling using the PetroMod software. Results show that the low-overpressure interval is mainly composed of sandstone sediments. The porosities of sandstone in the low-overpressure interval primarily range from 15%-20%, and the permeabilities range from 10–100 md. Analysis of the geochemical parameters of C1, iC4/nC4, ΔR3, and numerical modeling shows that oil and gas migrated upward into the sandstone in the low-overpressure interval, and then migrated along the sandstone of low-overpressure interval into the Yacheng uplift. The low-overpressure both underlain and overlain by overpressure resulted from the fluids migrating along the sandstones in the low-overpressure interval into the Yacheng uplift since 1.9Ma. The mudstone in the strong overpressure interval is good cap overlain the sandstone of low-overpressure interval, therefore up-dip pinchouts or isolated sandstone in the low-overpressure interval locating the migration path of oil and gas are good plays for hydrocarbon exploration.

## Introduction

Overpressure is an obvious geologic feature in some petroliferous basins and have been estimated using well data [1–2]. There exists a widespread strong overpressure in the middle-deep Formation (2900–5000 m) of the Qiongdongnan Basin, and the pressure coefficient can be up to 2.27 (the strong overpressure refers to that pressure coefficient is greater than 1.8) [3–6]. Overpressure studies in the Qiongdongnan Basin show that overpressure retards organic-matter maturation and petroleum generation [7,8], and drives natural gas accumulation in the Ya13-1 Gas Field [9,10]. Overpressures (achieved by direct pore pressure measurements) in the Qiongdongnan Basin are associated with anomalously high porosity, compared with normally pressured sediments for a given depth [11].

The drilling analysis shows that there exists a composite configuration where the overpressure increases with depth at the upper interval, decreases at the middle interval, then increases again at the lower interval [3,6,12]. The middle decreasing overpressure in this composite configuration is called the low-overpressure interval in this paper. The low-overpressure interval primarily lies in the Neogene strata of the Yanan sag [6].

The low-overpressure interval between the strong overpressure intervals was also found in the Huanghua depression of the Bohaiwan Basin, China [13]. The causes of the low-overpressure interval between the strong-overpressure intervals in these basins are not well understood. The low-overpressure interval in the Qiongdongnan Basin was called the release interval of overpressure [6]. This implies that the low-overpressure interval originated from the overpressure release along the sandstone, however, the evidences were not showed in his paper. Many papers show that faults, fractures, diapir and connected sandstone are main conduits of overpressure release [14–20].

The objectives of this article are to (1) analyze the characteristics of the low-overpressure distribution based on test pore pressure data, well-log, seismic data and modeling data; (2) investigate the generation mechanism of the low-overpressure interval in the Qiongdongnan Basin based on the geophysical and geological data.

## Geological background

**Fig 1:**
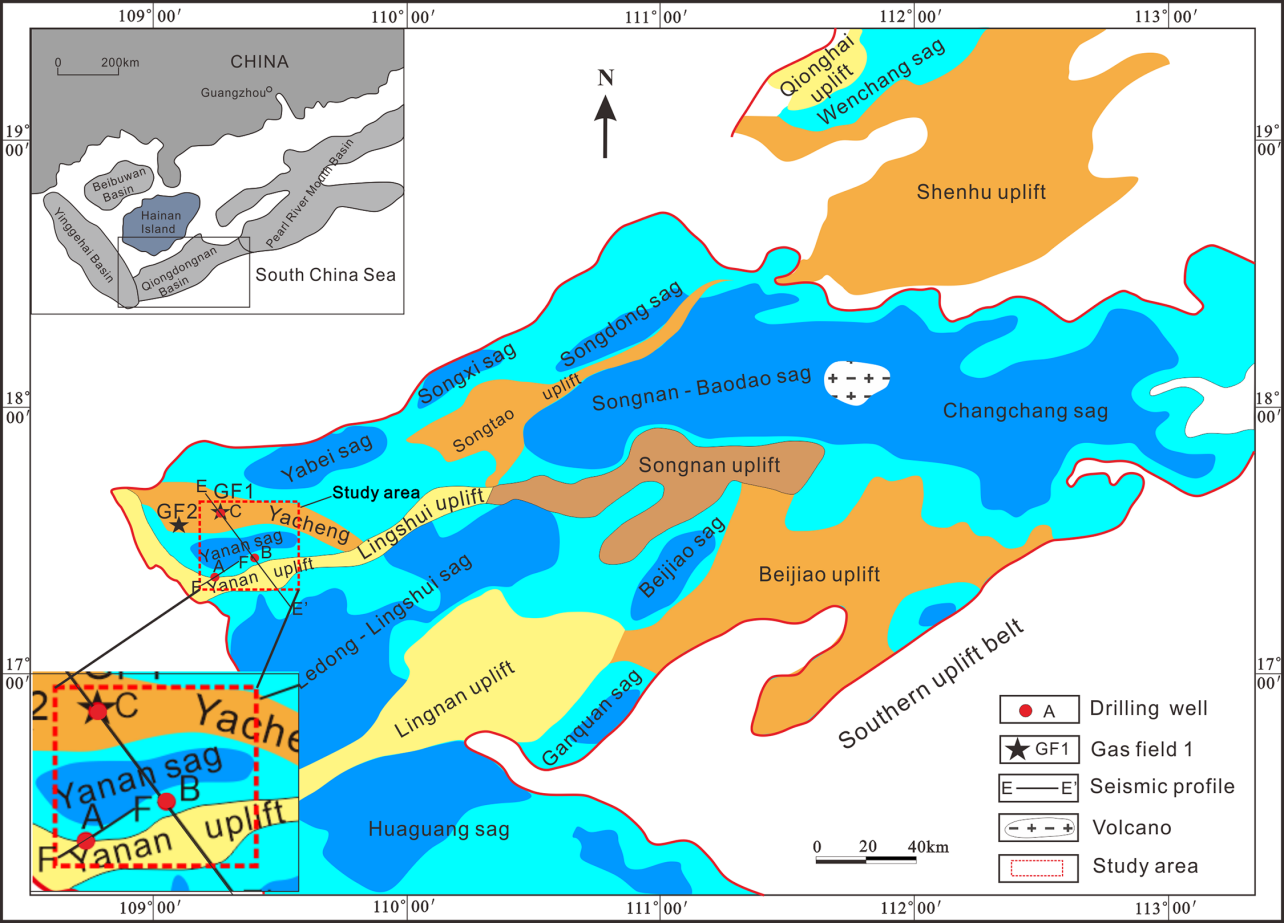
Structural units of Qiongdongnan Basin and the location of gas field, wells and seismic profiles (modified after Zhu, 2007).

The Qiongdongnan Basin is located in the northern part of South China Sea (108°50′– 111°50′E, 16°50′– 19°00′N), trending east-northeast (Fig 1). The basin covers an area of about 45 000 km^2^ [3]. The maximum thickness of Cenozoic sediments in the basin is in excess of 12 000 m. It can be divided into eight sags, namely the Yanan, Yabei, Songxi, Songdong, Ledong-Lingshui, Songnan-Baodao, Changchang, and Beijiao sags (Fig 1). The basin underwent rifting from 50.0 to 21.0 Ma, thermal subsidence from 21.0 to 10.5 Ma, and then rapid subsidence from 10.5 Ma to the present [21]. The break-up unconformity of T60 (21.0 Ma) divided the Cenozoic formations into two tectonic parts. Faults are primarily distributed in the tectonic part of the region from the basement to the T60 horizon; the faults are seldom active in the tectonic part of the region above the T60 horizon.

**Fig 2:**
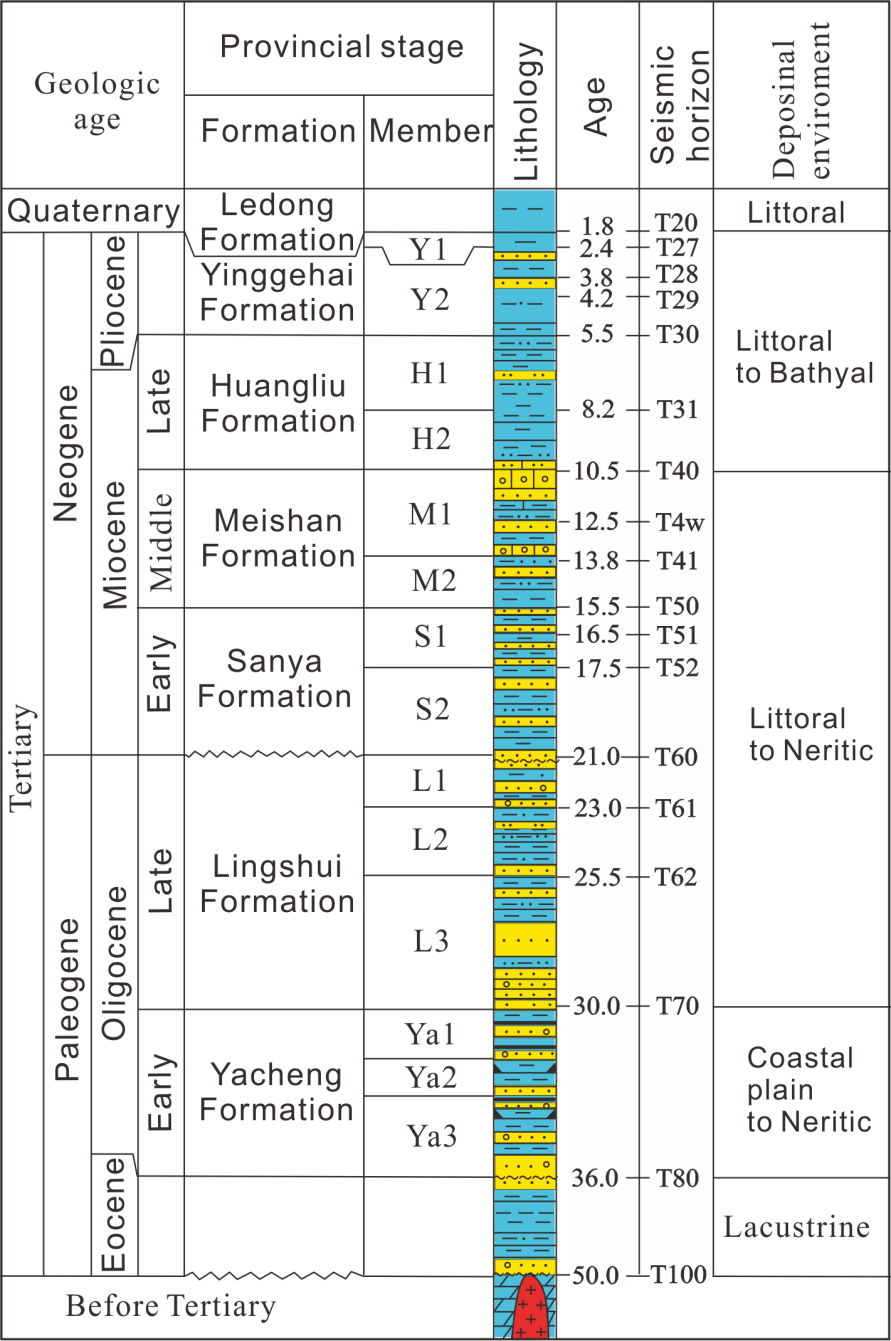
Generalized stratigraphic column for Qiongdongnan Basin (from Zhu, 2007; Shi et al., 2013).

The Qiongdongnan Basin is filled with Eocene and Oligocene rift sediments and Miocene-Quaternary postrift sedimements [8]. The Eocene sediments and Yacheng, Lingshui, Sanya, Meishan, Huangliu, Yinggehai, and Ledong formations can be identified by geologic and geophysical data, from bottom to top (Fig 2). The basin has been filled as a result of both continental and marine sequences. Continental facies dominated in the Eocene, whereas marine facies are found from the Yacheng Formation to recent strata (Fig 2).

The study area is located in the Yanan Sag and Yacheng uplift (Fig 1). Several large oil and gas fields have been discovered in the shallow water area in the northern part of basin. However, exploration in the Qiongdongnan Basin is now gradually extending from shallow to deep water areas. Exploration in the deep water area indicates the petroleum potential is huge in the Qiongdongnan Basin.

## Data and method

This study employed three main approaches: (1) pore pressure test data and well-log analysis to confirm the presence of the low-overpressure interval between the strong-overpressure intervals, (2) seismic velocities used to predict the low-overpressure interval distribution, (3) numerical modelling to illustrate the distribution and evolution of the low-overpressure.

### Well-log responses to overpressure distribution

Overpressure refers to pore pressures greater than the corresponding hydrostatic pressure [30]. Pore pressure in sandstone can be directly measured by using repeat formation testing (RFT), modular dynamic testing (MDT) or a drill stem testing (DST). In permeable rocks, record fluid pressure is close to the actual pore pressure. Pore pressure in the mudstones is commonly estimated based on wire-line logging methods and analysis of drilling parameters because the pore-fluid pressure in the mudstones usually cannot be measured directly because of their low permeability [1]. However, a few pore pressure test data are not sufficient for an area overpressure analysis, so they are supplemented with electronic logs used to predict and analyze the pressure of wells because pore pressures in the seals and the associated reservoir rocks are commonly equal. P-wave sonic, resistivity, density and mud pressure logs can provide good indications of overpressure [22–23]. Overpressure in the Qiongdongnan Basin can be identified using the P-wave sonic, resistivity, density logs deviating from the normal compaction trend [3].

**Fig 3:**
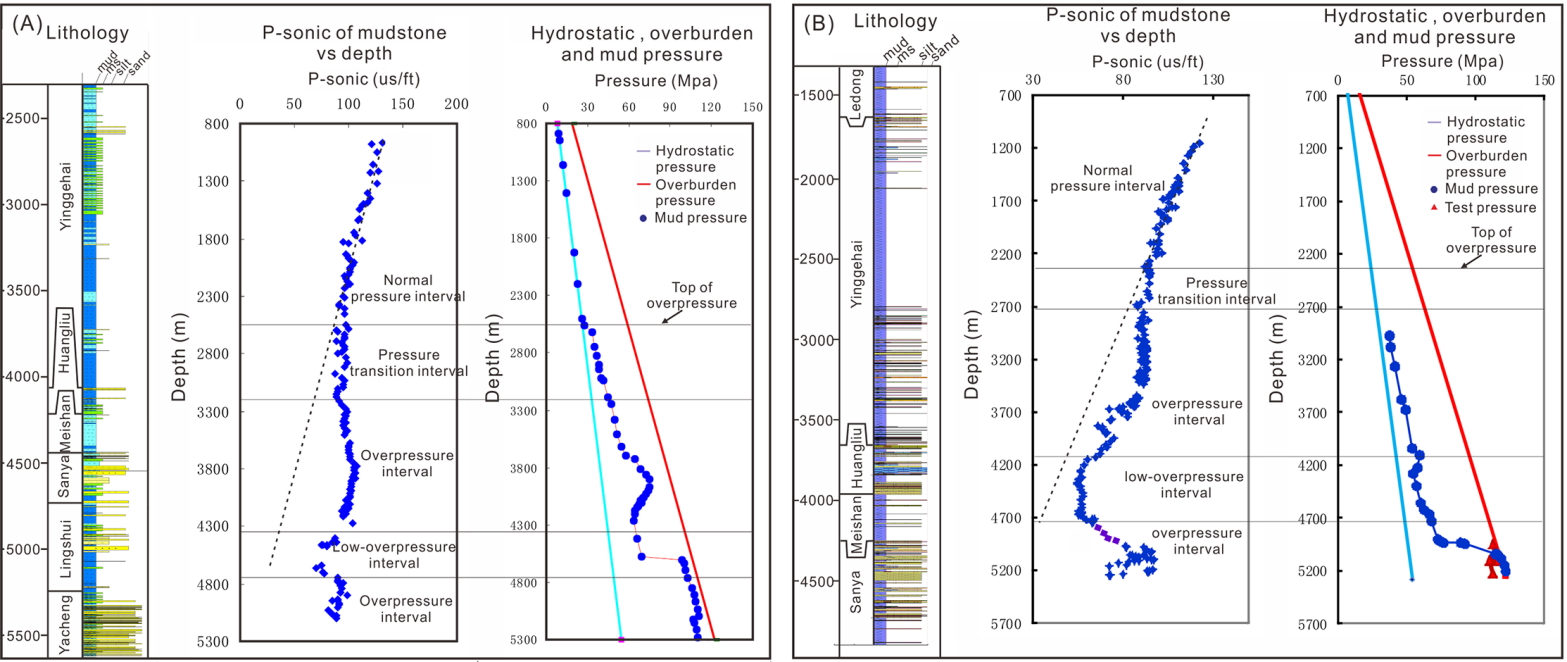
Sonic depth profiles of P-wave of mudstones and mud weights, as well as measured pressure coefficient data for wells A (panel A) and B (panel B) in the Qiongdongnan Basin. Locations of these two wells are shown in Fig 1. Sonic data are average values of single mudstone layers, and lithology is determined from cuttings. Test pressures are from DST (drill stem test).

Two representative wells located in the Yanan sag were selected to show the vertical distribution of low-overpressure interval (Fig 3). Fig 3 shows that the P-wave sonic for wells A and B deviates from the compaction trend and increase below about 2650 and 2360 m, respectively. The calculated mud weight and well testing pressure show that there is an overpressure below 2650 m and 2360 m in wells A and B, respectively. Intervals of normal pressure, pressure transition, low-overpressure and overpressure can be identified on logs and in the mud pressure profile. The low-overpressure interval lies between the strong overpressure intervals where the pressure coefficients for well B measured by drill stem test (DST) are 2.21, 2.2 at the depth of 4943 m and 5092.5 m, respectively (Fig 3B). The low-overpressure intervals for wells A and B primarily lie in the first member of the Sanya formation and Meishan formation.

### Pressure prediction based on the seismic velocity

Pore pressure prediction is critical in hydrocarbon exploration [24] and is especially important in Tertiary Qiongdongnan Basin where all economic fields exhibit overpressures [8,25]. The technique of using a decrease in seismic velocity to predict overpressure has been widely used since the pioneering work of Pennebaker [26]. Since then, seismic velocity has remained a main way to predict overpressure in the extensional basin [26–32]. Because there are limited DST, RFT, MDT measurements of pore pressure, therefore the technique of using a decrease in seismic velocity to predict overpressure has been used in the Qiongdongnan Basin, and it has a good correlation between an overpressured section and the deviation of the velocity in the Qiongdongnan Basin [4,5,6,33,34]. Fig 3 indicates that there is a good agreement between the overpressure and the deviation of the velocity, and that overpressure can be predicted by the deviation of the seismic velocity, so the method of Eaton (1972) was usually selected to calculate the overpressure [27]. The pore pressure is estimated using the equation

$$P_{p}=S_{v}-\left(S_{v}-P_{hyd}\right){\left(\Delta T_{n}/\Delta T_{\log}\right)}^{r}$$ (1)

Where P_p_ is the actual pore pressure, P_hyd_ is the normal hydrocarbon pore pressure, the subscripts n and log refer to the normal and measured values of sonic delta-t, r is an exponent.

**Fig 4:**
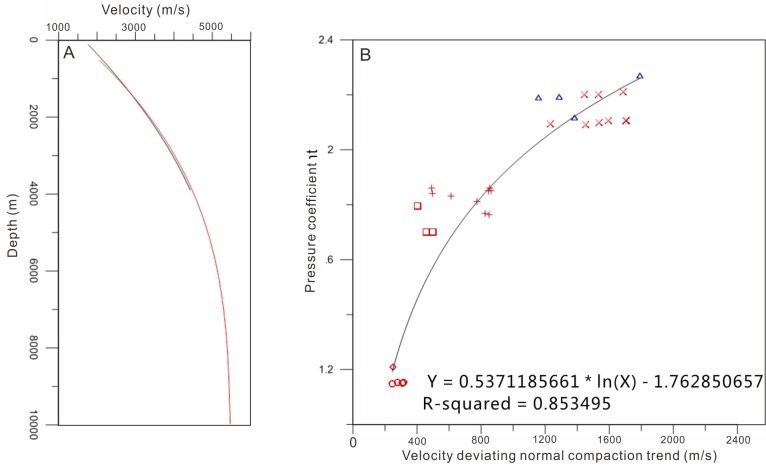
(A) Normal compaction trend constructed based on the P-velocity data from wells characterized by the normal pressure (blue line) and the interval velocities calculated from the stacking velocities (red line). (B) Relationship between the velocity deviating normal compaction trend and measured pressure coefficient of wells in the Qiongdongnan Basin.

**Fig 5:**
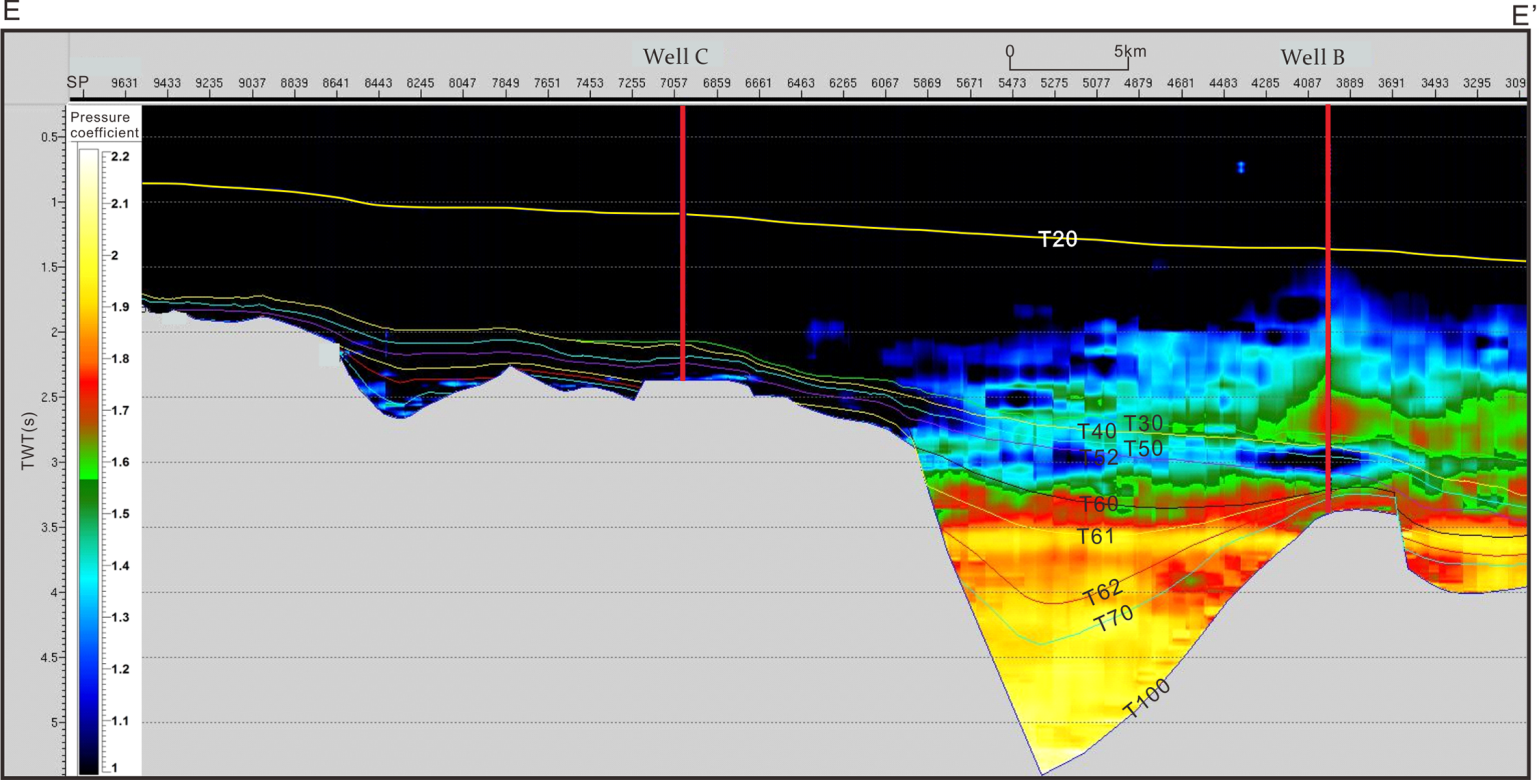
Pressure coefficient profile and interpreted low-overpressure interval of cross section E-E’, which shows that there exist a low-overpressure interval between the strong overpressure intervals in the Yanan sag of the Qiongdongnan Basin. The cross section location is shown in Fig 1.

However, there is limited actual pore pressure measured in study area, but the pressure coefficients measured by DST data. Therefore, we used the deviation of velocity to calculate the pressure coefficient. Firstly, the deviation of the velocity from the normal compaction velocity needs to be estimated. The normal trend velocity starting from the seabed was created based on the P-velocity data from the wells characterized by the normal pressure (blue line) and the interval velocities calculated from the stacking velocities (red line) (Fig 4A). Therefore, the deviation of the velocity from normal compaction velocity can be calculated. Pressure coefficients measured by DST data were selected to fit the relationship between the deviation of the velocity and the pressure coefficients (Fig 4B). The correlation coefficient between these two parameters can reach 0.85 (Fig 4B). According to this relationship, a pressure coefficient profile was then calculated for profile E-E’ (Fig 5). Fig 5 shows that there exist low-overpressure intervals with pressure coefficient 1.1–1.4 between the top strong-overpressure intervals with pressure coefficient 1.5–1.9 and the bottom strong-overpressure intervals with pressure coefficient 1.8–2.0. The low-overpressure intervals disappear northward in Yacheng uplift, and that lie in the Sanyan and Meishan formation in accordance with the sequences drilled by wells.

### Pressure evolution modelling

In order to understand pressure evolution, 2D modeling was carried out at the same profile E-E’ using the PetroMod software. The PetroMod is a finite element basin simulator which can simulate 2D and 3D hydrocarbon generation, migration and accumulation in sedimentary basins. This software can also be used to simulate pore pressure history and hydrocarbon migration in the extensional basin. The Qiongdongnan basin belongs to the extensional basin. Two different assumptions are used to calculate the pore pressure field. Firstly a special mass balance is considered which takes the compactible and deformable properties of the rock and the fluid (water) into account. Secondly compaction induced water flow is assumed to be so slow that water as a Newtonian fluid can described by Darcy's law. The pore pressure can be regarded as the driving forces of migration, and the transport properties depend on the permeability and viscosity values.

**Fig 6:**
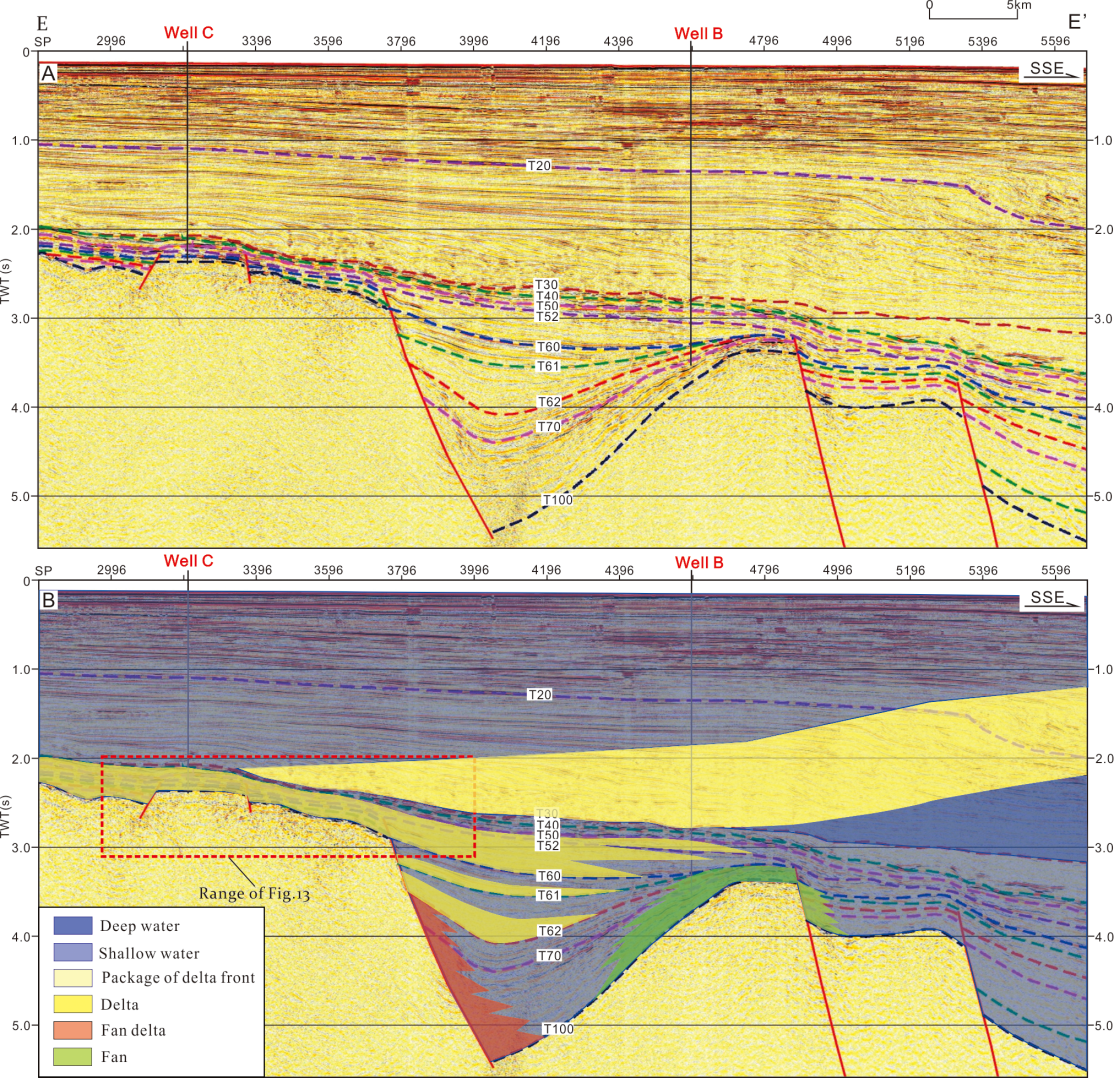
(A) Seismic profile and its depositional facies of cross section E-E’. (A) Seismic cross-section of profile E-E’. (B) Depositional facies of profile E-E’ indicating a lithological frame in the modeling. The cross section location is shown in Fig 1.

**Fig 7:**
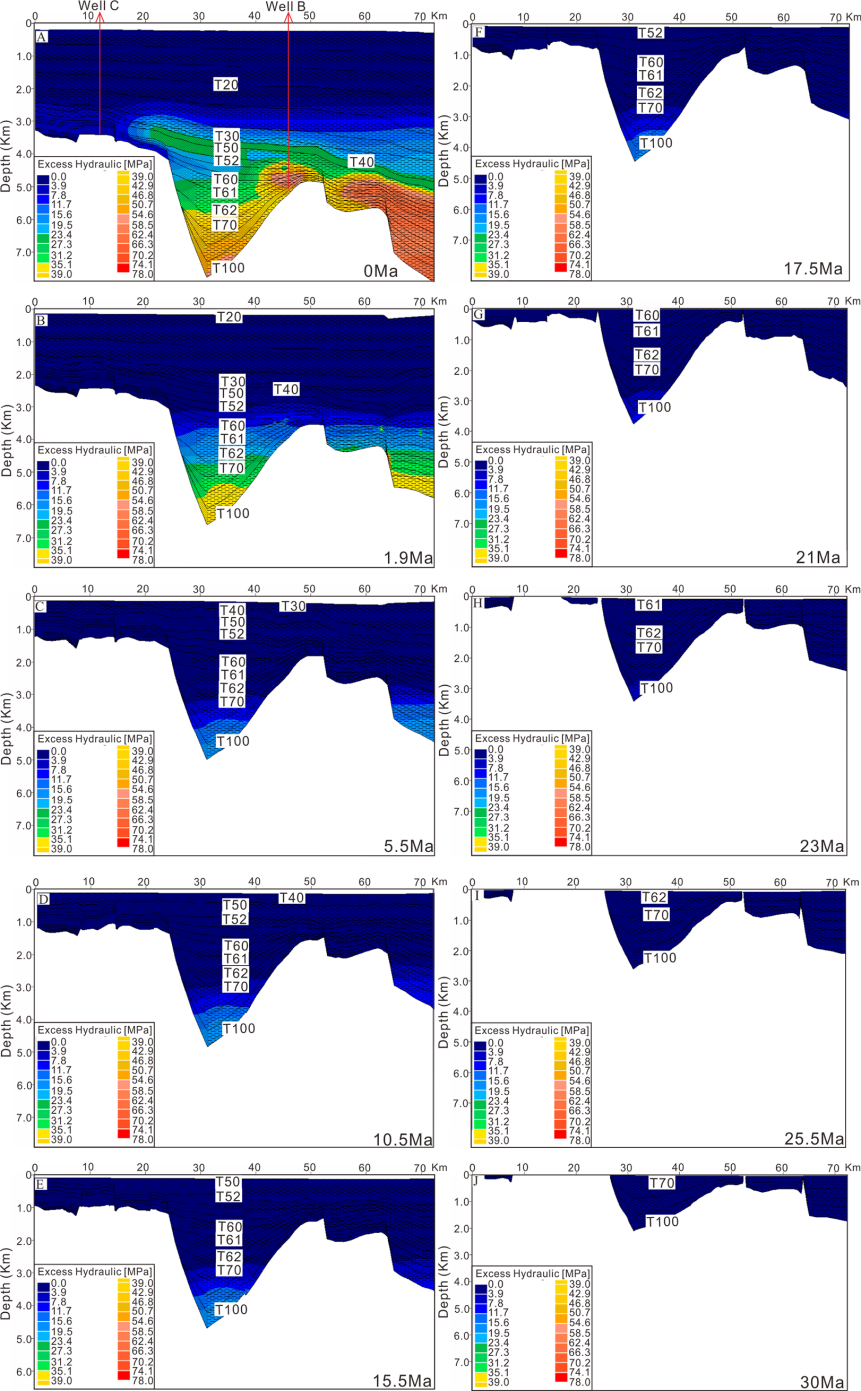
Excess hydraulic pressure of profile E-E’ in the different periods. The low-overpressure interval happened since 1.9Ma. The cross section location is shown in Fig 1.

The input data for the 2D modelling include age, lithology, erosion thickness, kerogen type, TOC, HI, faults activity, heat flow and paleo water depth. Most of these parameters can be derived from the test data of wells and research results [21]. Some of these parameters such as age, paleo water depth are listed in Fig 2. The parameters of kerogen type, TOC and HI were derived from the test data of wells. The strata lithology was assigned according to the statistics of drilled lithology and the lithological frame listed in Fig 6B. Primary erosion in the Qiongdongnan Basin occurred in the late Oligocene and the erosion thickness is about 340m in the area of wells B and C [21]. Faults activity can be identified by analyzing the faults distribution and comparing the Formation thickness between footwall block and hanging wall block. Fig 6 shows that faults are active in 50–21 Ma. Heat flow in the Qiongdongnan Basin is 58.7–87.1 mW/m2, and tends to increase from the continental shelf to continental slope owing to the lithosphereic/crustal thinning in the Cenozoic [35]. Measured temperatures, due to their direct relationship to thermal gradients and thermal conductivities, can therefore be used to constrain heat flow for a given set of thermal conductivities and measured temperatures. However, measured temperatures are only sensitive to thermal perturbations in the relatively recent geological past, and can therefore only be used to calibrate the recent heat flow values. The calibration of longer-term thermal processes, i.e. paleotemperatures or paleo-heat flow, is possible by using organic material which records the effects of temperature through geologic time as maturity indicators. The most commonly used indicator is the reflectance of the vitrinite maceral in coal, coaly particles, or dispersed organic matter. The modeled results show that there has develped low-overpressure interval since 1.9Ma in the Sanya and Meishan formations of the Yanan sag (Fig 7), and that there is same low-overpressure interval confirmed by the pressure prediction and pressure modelling (Figs 5 and 7).

## Results and discussion

### Correlation between the rich-sandstone strata and the low-overpressure interval

**Fig 8:**
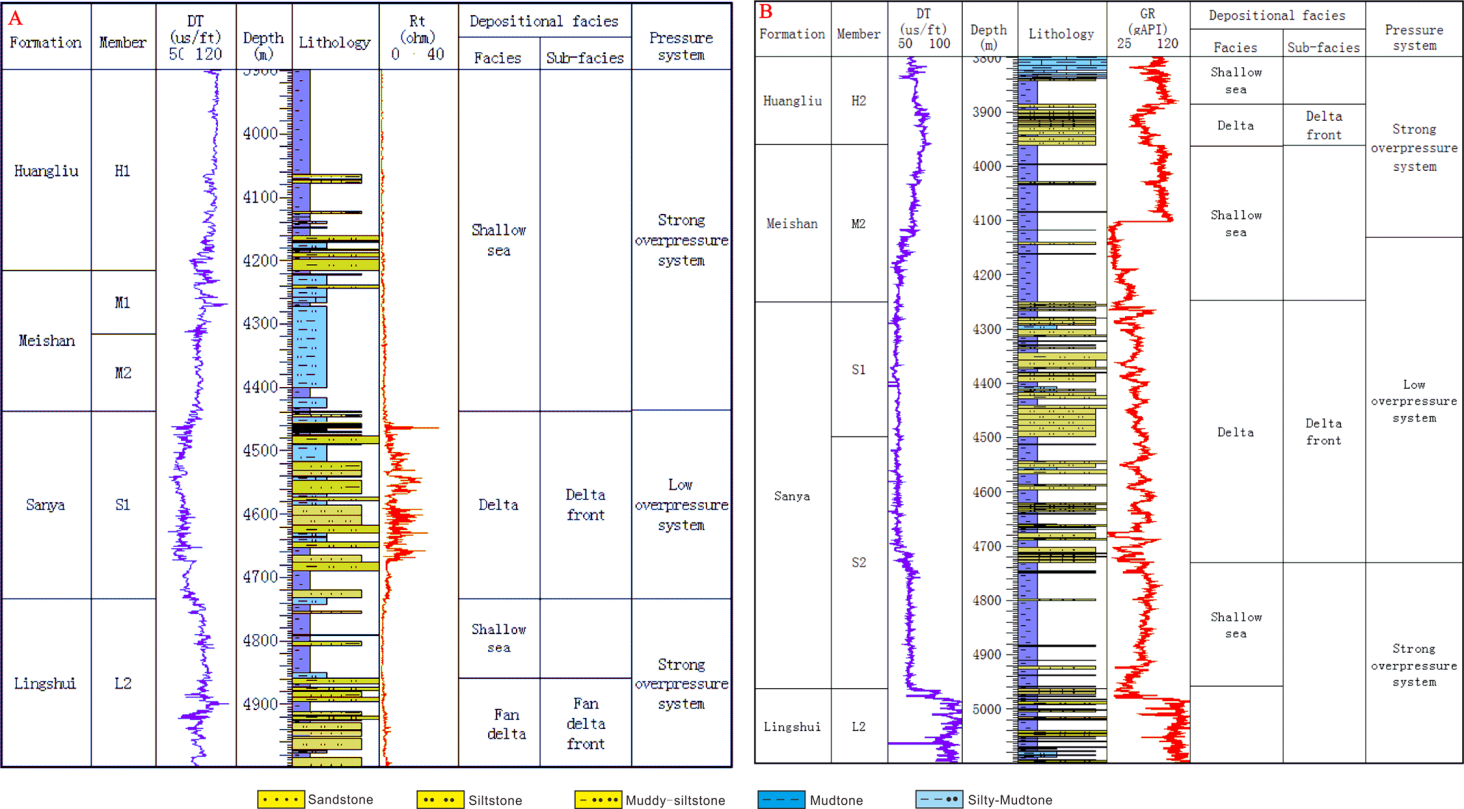
Generalized columns of well A and B. (A) The low-overpressure interval of well A at the depth of 4436-4733m correlate with the siltstone deposited by the delta front in the S1 sublayer of the Sanya Formation. (B) The low-overpressure interval of well B at the depth of 4131-4730m correlate with the sandstone deposited by the delta front in the S2 sublayer of the Sanya Formation and the mudstond deposited by the shallow sea in S1 sublayer of the Sanya Formation.

**Fig 9:**
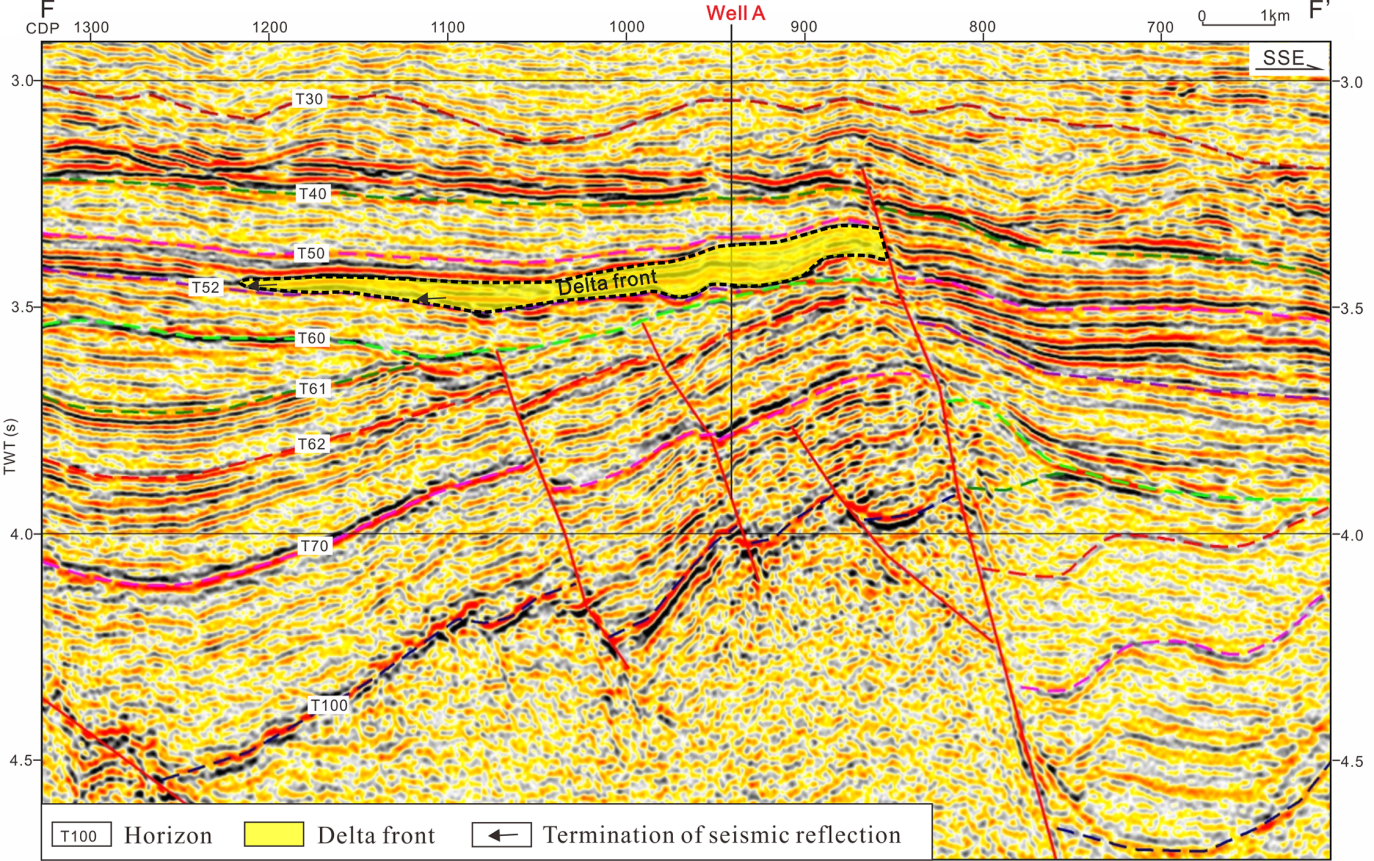
Seismic profile showing the delta front sediments which correlate with the low-overpressure interval drilled by well A.

Overpressure can release through faults, fractures, widespread sandstone and diapir systems [17–20]. The low-overpressure interval between the strong-overpressure intervals has been identified based on the well data, pressure prediction and numerical modelling in the study area (Figs 3, 5 and 7). In order to analyze the lithology in the low-overpressure interval, generalized columns of wells A and B was created based on the lithology, electric logs and pressure analysis shown in Fig 3. Fig 8A shows that the low-overpressure interval of well A correlates with the rich-siltstone strata deposited in the S1 sublayer of the Sanya formation, and that the strong-overpresure interval in the S2 sublayer of the Lingshui Formation correlates with mudstone and rich-siltstone sediments. Fig 8B shows that the low-overpressure interval of well B correlates with the rich-sandstone strata deposited by the delta front in the Sanya Formation and the mudstond deposited by the shallow sea in the bottom of the Meishan Formation, and that the strong-overpresure intervals correlate with the rich-mudstone strata deposited by shallow sea. The delta front drilled by well A in the S1 sublayer of the Sanya formation was interpreted as widespread sediments based on the seismic facies (Fig 9). Well B drilled the widespread delta front in the Sanya Formation (Figs 6B and 8B). These indicate that not all rich-sandstone sediments correlate with the low-overpressure interval such as the L2 (Fig 8A), but the low-overpressure interval usually compose widespread rich-sandstone sediments such as S1 (Fig 8).

An issue is how to understand that 115m mudstone of well B in the bottom of the Meishan Formation correlates with the low-overpressure interval (Fig 8B). Because the lithology of mudstones in the Meishan is similar to that in Sanya Formation [16], the resonable explanation is that the pore fluids in the 115m mudstone drived by the overlying overpressure in the mudstone released into the underlying widespread sandstone deposited by delta front in the process of compaction and overpressure generation (Figs 6B and 8B), and that the released fluids migrated along the sandstone deposited by the delta front and keep the low-overpressure becasued of it’s huge accomdation.

### Porosity of the sandstones in the low-overpressure interval

**Fig 10:**
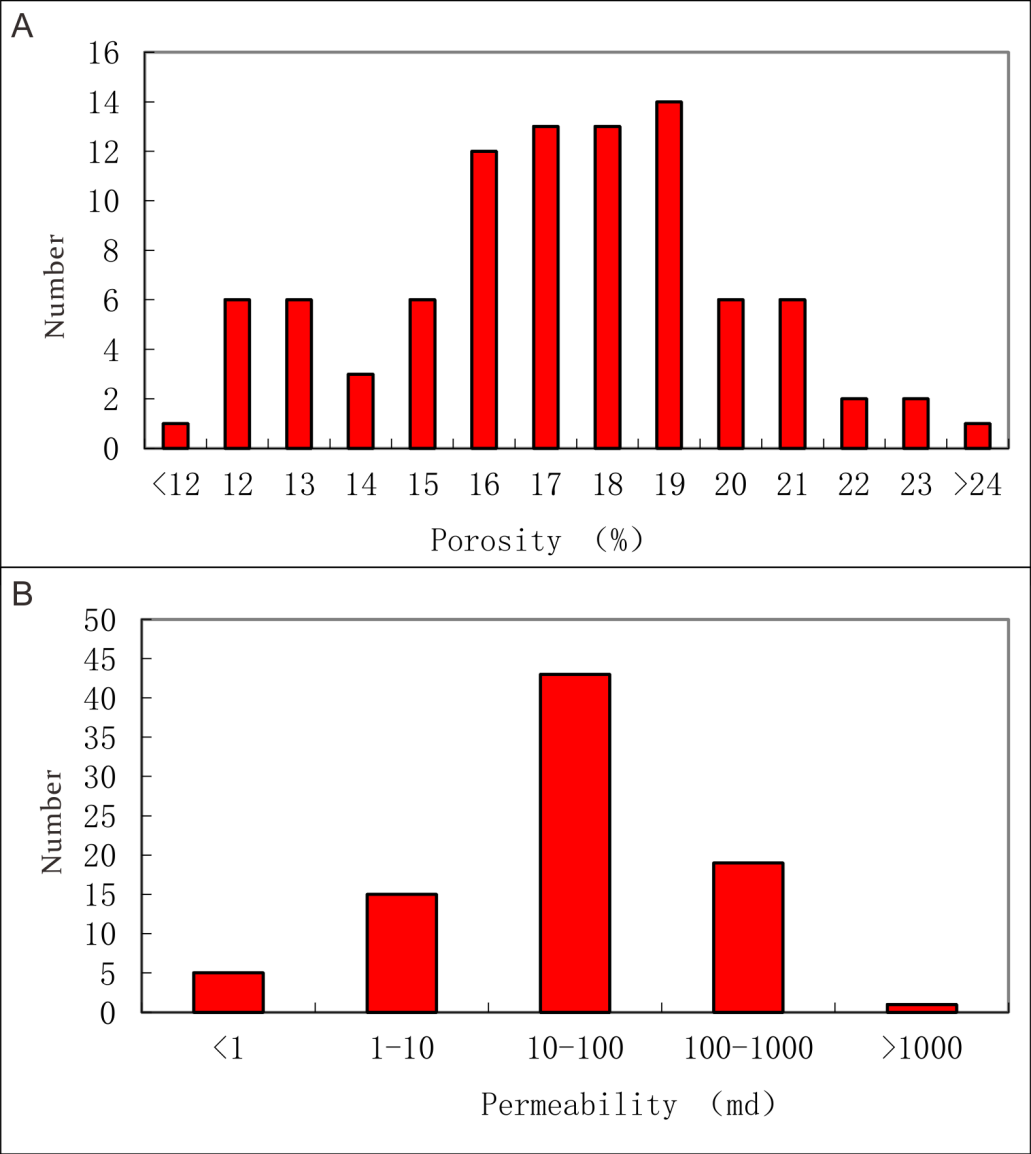
Porosity and permeability distribution in the low-overpressure interval showing a good migrated ability.

Releasing efficiency of the overpressure through sandstone is dependent on the porosity and permeability of sandstone in the low-overpressure interval. The sandstone characterized by high porosity and permeability is with high efficiency for fluids migration and overpressure releasing. We collected 91 samples of porosities and permeabilities measured for sandstone in the low-overpressure interval to investigate the porosity and permeability distribution. Fig 10A shows that porosities of the sandstone in the low-overpressure interval range from 12%-24%, but that most of porosities range from 15%-20%. Fig 10B shows that the permeabilities of the sandstone in the low-overpressure interval range from 1–1000 md, but most of permeabilities range from 10–100 md. The distribution characteristics of porosities and permeabilities indicate that overpressure can release efficiently through the sandstone in the low-overpressure interval.

### Geochemical parameters indicating the overpressure releasing through the sandstone in the low-overpressure interval

Much research shows that the combination of geochemical parameters can be used as a good indicator for natural gas migration [7,36,37,38]. Because gas components are obviously different in ways of mass fraction, molecule structure, solubility and absorbability, the fractionation phenomena that methane migrate ahead the heavier gaseous hydrocarbons, normal butane and isobutene will happen after nature gas migrate through water-saturated sediments, the fractionation phenomena can be used to indicate the gas migration [36,37,35].

**Fig 11:**
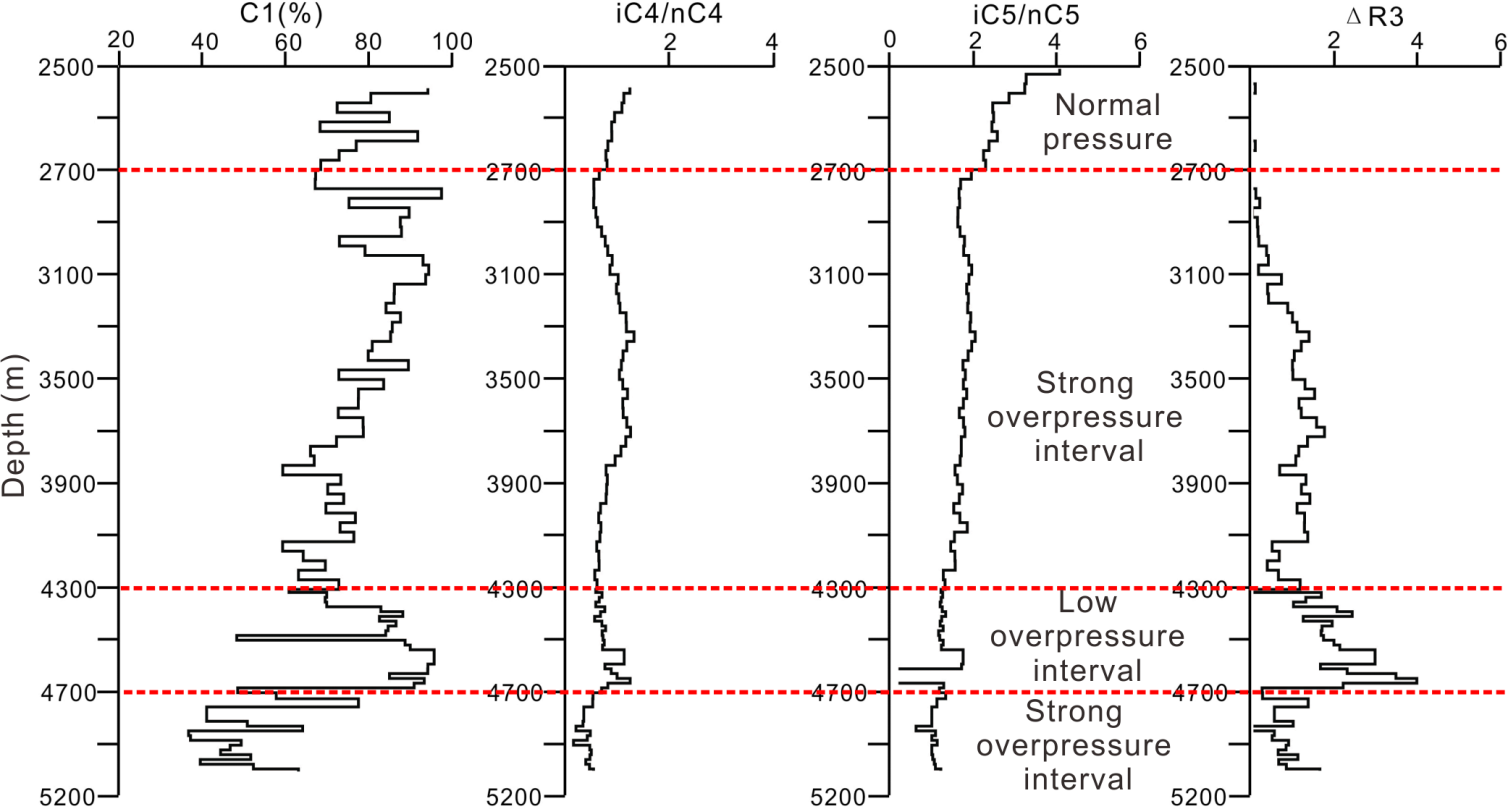
Geochemical parameters show that C1, iC4/nC4 and ΔR3 increase significiently in the low-overpressure interval, and that the three parameters gradually decrease upward in the low-overpressure interval. These indicate that the nature gas migrated upward into the low-overpressure interval, and then migrated along the sandstone of the low-overpressure interval.

According to the fractionation phenomena for gas components, we collected the test data about gas components of Well B to investigate the gas migration in the low-overpressure interval. In this paper, four geochemical indicative parameters, C1(%), m(iC4)/m(nC4), m(iC5)/m(nC5)and ΔR3 {ΔR3 = (R3–R4)/R4 = (m(iC4)/m(nC4)-m(iC4)/m(nC3)}/{m(iC4)/m(nC30}, were selected to investigate the gas migration characteristics by analyzing the vertical change of these four parameters. Fig 11 shows that the parameters of C1, iC4/nC4 and ΔR3 increase significantly in the low-overpressure interval, and that the three parameters close to bottom of the low-overpressure interval is bigger than that close top of the low-overpressure interval. These indicate that the fractionation phenomena happened in the low-overpressure interval, and that the nature gas migrated from lower strong overpressure interval upward into the low-overpressure interval, and then migrated along the sandstone of the low-overpressure interval.

**Fig 12:**
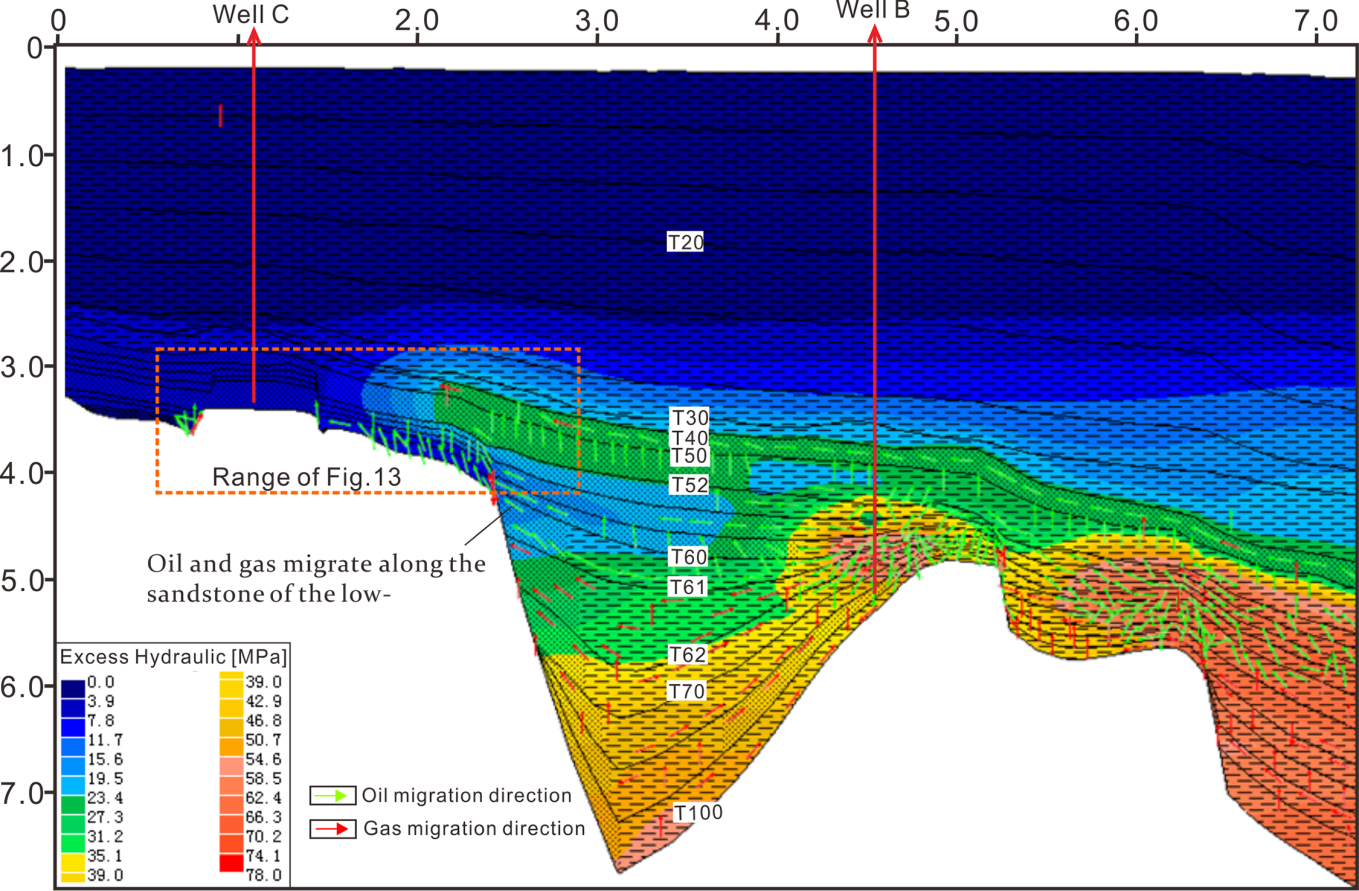
Numerical modeling shows that oil and gas migrated upward into the low-overpressure interval and migrated along the sandstone of low-overpressure interval into the Yacheng uplift.

In order to investigate further the oil and gas migration characteristics, the oil and gas generation and migration was modeled together with the pressure evolution based on the same parameters (Fig 12). Fig 12 shows that Oil and gas migrated upward into the low-overpressure interval, and then migrated along the sandstone in low-overpressure interval into the Yacheng uplift. These have a good agreement with the analysis results indicated by the Fig 11. A conclusion can be drawn that the generation mechanisms of the low-overpressure result from the fluids migrating along the sandstone of the low-overpressure interval into the Yacheng uplift, and that fluids migration leading to overpressure releasing in the low-overpressure interval. The modeled result shows that the low-overpressure interval generated since 1.9Ma in the Yanan sag (Fig 7), indicating the overpressure release and fluids migration happened since 1.9Ma.

### Implication for hydrocarbon exploration

**Fig 13:**
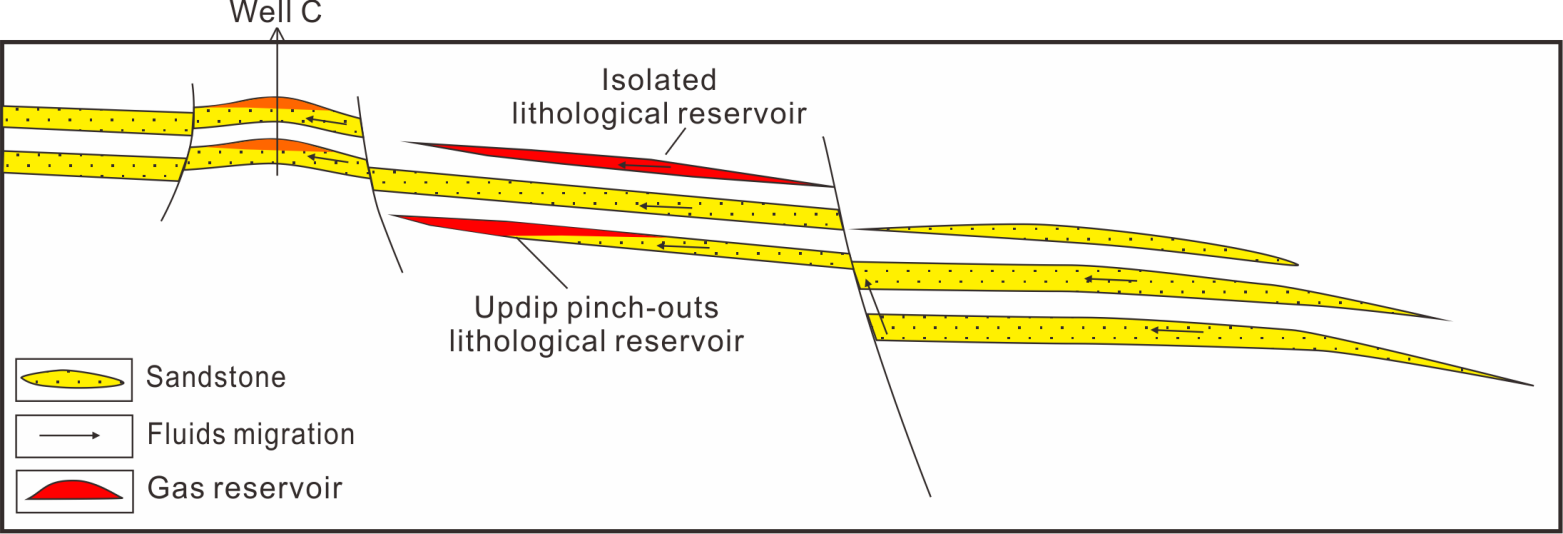
Schematic diagram of the lithological gas reservoirs. Updip pinch-outs or isolated traps in deltas locating in the migration paths of oil and gas from the low-overpressure interval and faults are good plays for hydrocarbon exploration.

Previous results show that the fluids migrating along the sandstones of the low-overpressure interval into the Yacheng uplift. The well C drilling shows that there exists a big gas field in the anticline of well C locating the migration path of oil and gas from the low-overpressure interval and faults (Figs 5, 6 and 12). Drilling confirms further that the oil and gas driven by overpressure migrated along the sandstones in the low-overpressure into the Yacheng uplift. Fig 6B shows that multi-delta deposited at the Yacheng uplift and Yanan Sag. Updip pinch-outs or isolated sandstone in deltas can form lots of lighological traps (Fig 13). These lithological traps locate in the migration paths of oil and gas from the low-overpressure interval and faults, so they are good plays for hydrocarbon exploration.

## Conclusions

Based on the above analysis, the following conclusions can be drawn:

1. The low-overpressure interval between the strong overpressures can be identified by the P-wave sonic and the mud weight in that all overpressured mudstones have higher P-wave sonic compared with normally pressured mudstones at a given depth.
2. The low-overpressure interval can be well predicted using the method of “normal compaction trend” based on a calibrated relationship between seismic interval velocities and pressure coefficients from wells.
3. The numerical modeling results using the PetroMod software show that there exists a low-overpressure interval confirmed by the drilling and pressure prediction. The low-overpressure interval generated since 1.9 Ma.
4. There is a good correlation between the sandstone sediments and the low-overpressure interval. The porosities of sandstone in the low-overpressure interval primarily range from 15%-20% and the permeabilities range from 10–100 md. Analysis of the geochemical parameters of C1, iC4/nC4 and ΔR3 and numerical modeling shows that oil and gas migrated upward into the sandstone in the low-overpressure interval, and then migrated along the sandstone of low-overpressure interval into the Yacheng uplift.
5. The generation mechanisms of the low-overpressure result from the fluids migrating along the sandstone of the low-overpressure interval into the Yacheng uplift since 1.9Ma. Updip pinch-outs or isolated sandstone of the low-overpressure interval locating the migration path of oil and gas are good plays for hydrocarbon exploration.
