# Expression of Concern: Identification of low-overpressure interval and its implication to hydrocarbon migration: Case study in the Yanan sag of the Qiongdongnan Basin, South China Sea

**DOI:** 10.1371/journal.pone.0349165

**Published:** 2026-05-12

**Authors:** The PLOS One Editors

The *PLOS One* Editors issue this Expression of Concern for this article [1] because of concerns identified about the peer review process. Readers are advised to interpret the article [1] with caution.

In addition, contrary to the declaration in the Data Availability statement, the original raw data files supporting the article’s results were not provided with the article, and the authors have not provided these data following editorial request. As the original raw data files supporting the article’s results have not been made available, this article [1] does not comply with the PLOS Data Availability policy.
